# Spatial Genetic Structure and Mitochondrial DNA Phylogeography of Argentinean Populations of the Grasshopper *Dichroplus elongatus*

**DOI:** 10.1371/journal.pone.0040807

**Published:** 2012-07-30

**Authors:** Natalia Rosetti, Maria Isabel Remis

**Affiliations:** Departamento de Ecología, Genética y Evolución, Facultad de Ciencias Exactas y Naturales, Universidad de Buenos Aires, Buenos Aires, Argentina; Biodiversity Insitute of Ontario - University of Guelph, Canada

## Abstract

Many grasshopper species are considered of agronomical importance because they cause damage to pastures and crops. Comprehension of pest population dynamics requires a clear understanding of the genetic diversity and spatial structure of populations. In this study we report on patterns of genetic variation in the South American grasshopper *Dichroplus elongatus* which is an agricultural pest of crops and forage grasses of great economic significance in Argentina. We use Direct Amplification of Minisatellite Regions (DAMD) and partial sequences of the cytochrome oxydase 1 (COI) mitochondrial gene to investigate intraspecific structure, demographic history and gene flow patterns in twenty Argentinean populations of this species belonging to different geographic and biogeographic regions. DAMD data suggest that, although genetic drift and migration occur within and between populations, measurable relatedness among neighbouring populations declines with distance and dispersal over distances greater than 200 km is not typical, whereas effective gene flow may occur for populations separated by less than 100 km. Landscape analysis was useful to detect genetic discontinuities associated with environmental heterogeneity reflecting the changing agroecosystem. The COI results indicate the existence of strong genetic differentiation between two groups of populations located at both margins of the Paraná River which became separated during climate oscillations of the Middle Pleistocene, suggesting a significant restriction in effective dispersion mediated by females and large scale geographic differentiation. The number of migrants between populations estimated through mitochondrial and DAMD markers suggest that gene flow is low prompting a non-homogeneous spatial structure and justifying the variation through space. Moreover, the genetic analysis of both markers allows us to conclude that males appear to disperse more than females, reducing the chance of the genetic loss associated with recent anthropogenic fragmentation of the *D. elongatus* studied range.

## Introduction

The evolutionary potential of a species depends mainly on the genetic variation of their populations, which is the consequence of a balance between evolutionary and demographic processes generating either heterogeneity or homogeneity among local populations. The amount of genetic variation within and among natural populations is mainly explained by several historical and contemporary processes, including genetic drift, effective migration, natural selection, fragmentation and range expansion [1]. Population genetics provides models and tools for interpretation of the processes that shape population structure. DNA based marker methods are usually used in ecological, evolutionary, and genetic approaches to analyse efficiently genetic structure in both animal and plant species [2], [3].

Since Jeffreys *et al.* [4], minisatellites have drawn the attention of researchers because they are highly variable and can potentially detect genetic variation within and between populations when other markers fail. Minisatellites, also called variable number of tandem repeats (VNTR), are tandem repeat DNA sequences that generally consist of 10–60 bp motifs. Most of the minisatellites share a common motif known as a core sequence [5]. Heath *et al*. [6] described a novel method termed direct amplification of minisatellite-region DNA (DAMD), which amplified regions that are rich in minisatellite repeats using PCR. This method uses the core sequence of minisatellites as a single primer, generating AFLP-like banding profiles [7].

Population genetic structure is not always mirrored in the geographical proximity of individuals. Therefore, identifying populations a priori can lead to errors due to potential biases caused by unidentified migrants and cryptic spatial structure [8]. Bayesian clustering methods use genetic information to assign individuals to populations without assuming predefined populations. They can assign either the individuals or a fraction of their genome to a number of clusters based on multilocus markers such as minisatellites.

One persistent challenge in the analysis of population genetic data is the need to account for the spatial arrangement of samples and populations. Spatial genetic structure is defined as the non-random distribution of genetic variation among individuals within populations. Unlike traditional genetic studies, landscape genetics incorporates tests to analyse the existence of probable landscape heterogeneity on gene flow and hence on genetic variation patterns [9].

Mitochondrial DNA (mtDNA) data have been extensively used to understand the spatial distribution of genetic lineages within species allowing the historical factor with the highest effect on the lineages spatial patterns to be identified [10]. Most relevant hypotheses in phylogeographical studies are related to dispersal and demographic processes, inferences which are usually based on estimates of genetic diversity, divergence time and demographic history [11]. Inferences concerning demographic events have become a central challenge in phylogeography, and the recent development of new, more objective mathematical models based on coalescent theory have enhanced the analysis of demographic history of natural populations. Modern genetic population approaches allow developing estimators of population parameters as the rate of mutation, the time of separation between two populations, the rate of migration between populations, the changes in population size as well as the action of natural selection [12], [13], [14].

Mitochondrial and nuclear markers differ in their mode of inheritance and in the rate of evolution. Mitochondrial sequences tend to have faster evolution with respect to nuclear sequences [15]. Markers with fast evolution tend to enhance recent demographic events; nonetheless, changes may saturate over long time periods [16]. Approaches comparing markers that evolve differently in time can offer complementary information to understand population structure.

Many grasshopper species behave as opportunist species, which are able to increase their population size and geographical distribution, in response to the expansion of cultivated areas. Most of them are considered of economic importance because of the damages they cause to crops and pastures [17]. Understanding population dynamic in environments altered by anthropogenic activity requires detailed information about genetic diversity and spatial structure of populations [18].

The grasshopper *Dichroplus elongatus* provides a helpful biological model to analyse the structure of populations that vary in their historical frequency in relation to control management strategies and the extent of demographic fluctuation because of their great biotic potential. *D. elongatus* is a South American grasshopper with a wide geographic distribution which has been reported as a dominant or codominant species in most grasshoppers outbreaks which have occurred in the last years [19]. Populations of this species have the ability to dramatically increase in size, making it an agricultural pest of crops (soybean and sunflower) and forage grasses of great economic significance [19], [20], [21].

Previous studies have revealed strong genetic structure at chromosome level. Population cytogenetic studies demonstrated that the persistence of B chromosomes in natural populations from Argentina may be explained mainly in a selective scenario. The maintenance on supernumerary chromosomes is the result of trade-offs among opposite selective effects and interactions with their mitotic instability [22], [23].

The efficiency of using the estimates of genetic diversity in the management of agronomically important species depends on the efficiency of detecting the population structure and identification of the origin of migrants [24]. The assessment of genetic diversity and population structure in *D.elongatus* samples using molecular neutral markers can gain insight into these aspects.

Our general goal is to analyse genetic variation in populations of this grasshopper species by DAMD, in order to generate a large number of characters needed for genomic scans, and by sequences of mitochondrial DNA. Our particular aims are to obtain complementary information to improve our understanding of the processes underlying the spatial and temporal dimensions of genetic variation and thereby reach a better idea of intraspecific structure, demographic history and gene flow patterns in 20 Argentinean populations of *D. elongatus* belonging to different geographic and biogeographic regions.

## Materials and Methods

### Sample Collection

A total of 385 adult males of *Dichroplus elongatus* (Orthoptera: Acrididae) was collected from 20 natural populations across three biogeographic regions (sense [25]) of Argentina (Table 1). No specific permission was required for these field studies because the grasshopper species studied did not involve an endangered or protected species.

**Table 1:** Locations and DAMD genetic diversity of 20 populations of *D. elongatus*.

BiogeographicRegion	Province	Population	Latitude	Longitude	N	*PLP*	*H_E_*	*I*	Symbol
Las Yungas	Tucuman	Raco	26°36’S	65°10’O	23	100	0.437	0.581	RCO
							(0.007)	(0.151)	
	Tucuman	Horco Molle	26°48’S	65°19’O	23	100	0.449	0.552	HCM
							(0.006)	(0.163)	
Espinal	Corrientes	Santo Tome	28°36’S	56°01’O	10	100	0.423	0.560	STO
							(0.008)	(0.197)	
		Yapeyu	29°28’S	56°49’O	21	100	0.426	0.601	YAP
							(0.007)	(0.153)	
		Monte Caseros	30°17’S	57°38’O	22	99.4	0.432	0.575	MCA
							(0.007)	(0.164)	
		Mocoreta	30°38’S	57°58’O	20	99.4	0.412	0.540	MOC
							(0.008)	(0.184)	
	Entre Rios	Concordia	31°24’S	58°02’O	20	99.4	0.415	0.555	CON
							(0.008)	(0.181)	
	Córdoba	Río Cuarto	33°08’S	64°20’O	19	97.4	0.414	0.545	RCA
							(0.009)	(0.184)	
		Río los Sauces	31°40’S	63°55’O	14	97.4	0.408	0.519	RLS
							(0.009)	(0.215)	
Pampeana		Santa Catalina	34°09’S	63°55’O	11	96.8	0.395	0.488	SCA
							(0.008)	(0.234)	
	Entre Ríos	Colon ER	32°13’S	58°09’O	19	98.7	0.419	0.567	COE
							(0.007)	(0.182)	
		Gualeguaychu	33°06’S	58°32’O	20	100	0.410	0.560	GUA
							(0.008)	(0.166)	
	Buenos Aires	Campana	33°59’S	58°57’O	22	98.1	0.422	0.534	CAM
							(0.008)	(0.202)	
		Carmen de Areco	34°49’S	59°50’O	20	97.4	0.411	0.547	CAR
							(0.009)	(0.188)	
		Cañuelas	35°03’S	58°46’O	19	98.1	0.399	0.566	CAÑ
							(0.008)	(0.179)	
		Punta Indio	35°16’S	57°16’O	16	98.7	0.409	0.537	PUI
							(0.009)	(0.188)	
		Las Flores	35°55’S	59°07’O	23	98.7	0.416	0.529	FLO
							(0.009)	(0.171)	
		Rauch	36°47’S	59°06’O	20	99.4	0.420	0.582	RAU
							(0.007)	(0.157)	
		Colon (BA)	33°52’S	61°05’O	21	97.4	0.398	0.543	COB
							(0.009)	(0.199)	
	Santa Fe	Venado Tuerto	33°45’S	61°57’O	21	97.4	0.408	0.550	VET
							(0.008)	(0.184)	

Name of biogeographic region, geographic province, geographical coordinates and abbreviations used throughout this paper are indicated for each location. Number of individuals (N), percentage of polymorphic loci (*PLP*), mean expected heterocygocity (*H_E_*), Shannon index (*I*). Standard errors are indicated in brackets.

### DAMD-PCR Method

Total genomic DNA was isolated according to the method of Marchant [26] with some modifications. Extracted genomic DNA was used as template DNA in a minisatellite repeat primed polymerase chain reaction (PCR), as described in Heath *et al*. [6]. Each reaction used one synthetic oligonucleotide design from a minisatellite repeat core sequence as a primer [6]. The utilised primers included M13 5′-GAG GGT GGC GGT TCT-3′ and INS 5′-ACA GGG GTG TGG GG-3′. Reactions were run using a touchdown approach with the first five cycles decreasing by 1°C per cycle from annealing temperatures of 50°C for INS and 51°C for M13 (to guarantee a high specificity), followed by 35 cycles with annealing temperatures of 46°C for INS and 47°C for M13 (60 s). We also used a 72°C extension cycle (90 s) and a 94°C denaturing cycle (60 s). Reaction mixtures (50 µl) contained 1X reaction buffer, 50 µM MgCl2, 200 µM dNTPs, 2 µM primer, 50 U/µl Taq polymerase (Invitrogen), and 200 ng DNA. PCR products (6 µl) were fractionated on 6% polyacrylamide gels for 3 h at 55 W, and the DNA bands were visualised by silver nitrate staining (Promega, Biodynamics). Bands ranging from 300 to 1,000 bp were scored for the further analysis.

### mtDNA Amplification and Sequencing

The polymerase chain reaction (PCR) was used to amplify a 526-base pair (bp) fragment of the mitochondrial gene cytochrome oxidase I (COI) in a total of 169 adult males using specific primers from [27]: D6 mtDNA 5′- GGAGGATTTGGAAATTGATTAGTTCC-3′ and D11 mtDNA 5′- ACTGTAAATATATGATGAGCTCA-3′. Each PCR was carried out in a total volume of 50 µl. PCRs contained 50 mM Cl_2_Mg, 50 µM dNTPs, 10 µM of each primer, 50 U/µl Taq polymerase (Invitrogen), 10 X buffer and 100 ng DNA template. The following temperature profile was used for the PCR: 94°C for 3 min, followed by 10 amplification cycles with an annealing temperature at 46°C for 1 min and 25 amplification cycles at 51°C for 40 s. Each cycle was preceded by a denaturalisation cycle at 94°C for 10 s and followed by an elongation cycle at 68° for 1 min. The final extension was conducted at 68°C for 10 min. After visualisation on a 1% agarose gel with a 100 bp ladder, PCR products were purified and sequenced on an ABI PrismTM Sequencer 31309l Genetic Analyzer (Applied Biosystems, Inc.) by the Macrogen INC., Seoul, Korea Sequencing Service.

### DAMD Statistical Analysis

The DAMD bands were scored as presence (1) or absence (0) and only those that were well defined in all populations were taken into account to generate the DAMD matrix dataset.

Independence of DAMD fragments were tested by means of the Fisher’s exact test with the Markov chain method considering 1000 iterations per batch and using GENEPOP software v4.0 [28].

### Intrapopulation Genetic Diversity

The genetic variability was estimated under two different conditions: by supposing Hardy- Weinberg equilibrium (*F_IS_* = 0) and by assuming *F_IS_* = 0.23 estimated previously through isoenzymatic analysis in Sequeira *et al*. [29]. *AFLPsurv* software [30] was used to estimate the percentage of polymorphic loci (*PPL*) at the 5% level and expected heterozygosity (*H*_E_) following Lynch and Milligan [31]. We estimated allelic frequencies at DAMD *loci* using the Bayesian method developed by Zhivotovsky [32] for diploid species.

In addition, the Shannon information index (*I*) [33] was calculated as a measure of gene diversity across all markers (loci) using POPGENE software [34].

### Genetic Differentiation among Populations and Gene Flow

Divergence among the sampled populations was assessed by an analysis of molecular variance (AMOVA, [35]) using GENEALEX software [36]. Due to the dominant expression of DAMD markers, the AMOVA analyses partitioned variation according to correlations among genotypes rather than variation in gene frequencies.

Genetic differentiation between pairs of populations was analysed through pair-wise *F_ST_* comparisons. The statistical significance of each of the variance components of the AMOVA and the paired comparisons was determined by nonparametric procedures using 1000 random permutations.

The gene flow between populations was estimated through *F_ST_* statistics according to Wright [37].

We also estimated population differentiation implementing a Bayesian approach for dominant markers without assuming Hardy-Weinberg equilibrium using HICKORY version 1.0.4. [38]. This Bayesian approximation estimated *θ^B^* and *f* coefficients, equivalents of *F_ST_* and *F_IS_* Wright indices, using Markov chain Monte-Carlo (MCMC) simulations. Results were examined under three probable models: i) full model (*f*≠0 and *θ^B^*≠0); ii) *f* = 0 model (*f* = 0 and *θ^B^*≠0); iii) theta  = 0 model (*f*≠0 and *θ^B^* = 0). Then, we compared models using the Deviant Information Criterion (DIC) [39].

Genetic structuring of the samples was investigated in parallel using 2 Bayesian population assignment methods implemented in STRUCTURE 2.1 [40] and BAPS 4.1 [41]. STRUCTURE treats the allele frequencies, the number of genetically distinct groups (K) in the sample and individual ancestry in each group as random variables to be simultaneously determined. The most likely partition of the data set was selected assuming correlated frequencies and admixture origin of populations, using 5 replicates with a 700000 repetition burn-in period and 500000 Markov chain Monte Carlo randomisations for each value in the range K = 1 to K = 20, with prior population information. The optimal K-value was chosen according to the maximum log-likelihood, L(K) output by STRUCTURE.

BAPS considers populations instead of individuals as sampling units and establishes populations with different allelic frequencies instead of partitioning individuals based on Hardy-Weinberg equilibrium. Under the spatial model, the population structure is estimated supposing as a priori information that the structure within a certain sampling area depends on the adjacent areas. Under the option “spatial clustering”, the population landscape is scattered into a “coloured Voronoi tesselation” [42]. We conducted admixture and mixture analysis of both individuals and populations with the geographic origin of the samples either used as prior information or not. BAPS was run with the maximal number of groups (K) set to 2–20. Each run was replicated six times, and the results were averaged according to the resultant likelihood scores.

### Phenetic Relationships

Nei’s genetic distances [43] were estimated among subpopulations by using the DAMD allele frequencies pooled within each population and the RAPDDIST subprogramme of the RAPD software [44]. Bootstrapping over loci was performed by using the RAPDBOOT subprogramme, with 1000 permutations. A consensus phenogram was estimated from the genetic distance matrix, using the NEIGHBOR and CONSENSE programmes of PHYLIP version 3.5 [45].

### Spatial Genetic Structure Analysis

We analysed the relationship between population pairwise genetic similarity and geographic distances based on the spatial autocorrelation multivariate approach development by [46] using GENEALEX software [47]. The 95% confidence intervals around r = 0 (under null hypotheisis of non spatial structure) were estimated by random permutations of all individuals among distance classes and the recalculation of r 1000 times to establish the upper and lower 95% confidence intervals around this value. Likewise we analysed the positive autocorrelation over short distances using a one-tailed test proposed by [48] estimating the level of significance (*P*) of observed r values though 1000 permutations. The 95% confidence intervals around each r value were estimated by bootstrapping. The correlation coefficient was plotted as a function of distance to produce spatial genetic correlograms.

We also performed Mantel tests [49] on the pairwise genetic and geographical distance matrices using the AIS software [50]. Detailed patterns of spatial genetic structure across the complete sampled area were visualised using the “genetic landscape shape” (GLS) interpolation procedure in AIS [50]. The design of this method allows to visualize patterns of genetic diversity across a surface in a three-dimensional space (*x*- and *y*-axes represent latitude and longitude whereas *z*-axis represents genetic distances). Genetic structure across the landscape was inferred from measured genetic distances using an inverse distance weighted interpolation across a uniform grid laid over the entire sampling area. A grid size of 60×60 was selected (we also tested 100×100 and 50×50 grids), with a distance weighting parameter (*α*) of 1.

### Mitochondrial DNA Statistical Analysis

Sequences were aligned using CLUSTAL 1.81 [51] and edited using BIOEDIT version 7.0.9 [52].

### Intrapopulation Genetic Diversity

The haplotypes were identified and characterised using the GENEALEX version 6.0 software [47].

Genetic variation was estimated using haplotype diversity *h* [53] and nucleotide diversity *π* [54] with the software ARLEQUIN version 3.5 [55].

### Phylogenetic Relationships among Haplotypes

Networks enhance haplotype relationships rather than conventional phylogenetic trees when recent multifurcations occur, the level of haplotype divergence is low and/or ancestral and derived haplotypes coexist [56]. Therefore, we also examined relationships among haplotypes with a reconstructed network in the NETWORK version 4.5 software [57]. We used the median joining algorithm with default settings for constructing the network (weight  = 10 and ε = 0).

**Table 2:** Population pairwise *F_ST_* statistics of 20 populations of *D. elongatus* (above diagonal).

	CAR	CAM	COB	VET	RAU	FLO	CAÑ	PUI	SCA	COE	GUA	RCA	RLS	MOC	CON	MCA	STO	YAP	RCO	HCM
**CAR**		0,130	0,099	0,097	0,123	0,145	0,143	0,116	0,188	0,122	0,150	0,164	0,168	0,151	0,126	0,109	0,136	0,085	0,102	0,122
**CAM**	1,673		0,177	0,167	0,130	0,181	0,159	0,174	0,206	0,131	0,162	0,144	0,194	0,189	0,174	0,135	0,156	0,143	0,138	0,161
**COB**	2,267	1,164		0,113	0,123	0,172	0,127	0,119	0,197	0,111	0,141	0,130	0,161	0,126	0,125	0,112	0,132	0,110	0,118	0,136
**VET**	2,324	1,245	1,968		0,115	0,164	0,123	0,152	0,216	0,131	0,142	0,135	0,156	0,163	0,133	0,100	0,118	0,103	0,128	0,136
**RAU**	1,781	1,676	1,784	1,923		0,090	0,121	0,116	0,166	0,099	0,087	0,132	0,143	0,130	0,103	0,110	0,100	0,087	0,110	0,136
**FLO**	1,473	1,129	1,200	1,275	2,514		0,168	0,154	0,203	0,160	0,119	0,191	0,153	0,144	0,118	0,141	0,138	0,128	0,128	0,131
**CAÑ**	1,503	1,321	1,718	1,775	1,813	1,234		0,145	0,145	0,100	0,137	0,092	0,122	0,117	0,104	0,089	0,085	0,079	0,096	0,148
**PUI**	1,901	1,184	1,850	1,395	1,912	1,378	1,477		0,130	0,139	0,140	0,178	0,147	0,146	0,151	0,125	0,109	0,117	0,105	0,161
**SCA**	1,080	0,961	1,018	0,909	1,253	0,979	1,473	1,677		0,169	0,168	0,174	0,164	0,205	0,149	0,164	0,175	0,117	0,152	0,202
**COE**	1,800	1,665	1,997	1,663	2,288	1,309	2,259	1,549	1,230		0,066	0,129	0,144	0,102	0,111	0,095	0,100	0,096	0,096	0,128
**GUA**	1,419	1,296	1,518	1,508	2,637	1,846	1,571	1,530	1,238	3,543		0,155	0,150	0,106	0,082	0,094	0,119	0,103	0,113	0,144
**RCA**	1,273	1,486	1,677	1,603	1,646	1,060	2,474	1,151	1,184	1,690	1,359		0,121	0,131	0,134	0,113	0,114	0,101	0,105	0,126
**RLS**	1,240	1,037	1,303	1,353	1,499	1,381	1,796	1,448	1,270	1,485	1,418	1,818		0,137	0,129	0,118	0,154	0,112	0,118	0,133
**MOC**	1,406	1,073	1,735	1,287	1,668	1,486	1,893	1,467	0,968	2,192	2,117	1,653	1,575		0,103	0,093	0,139	0,124	0,079	0,105
**CON**	1,736	1,185	1,745	1,634	2,188	1,862	2,159	1,406	1,429	2,003	2,794	1,610	1,683	2,185		0,101	0,119	0,077	0,115	0,147
**MCA**	2,053	1,607	1,974	2,255	2,017	1,525	2,562	1,750	1,273	2,379	2,404	1,968	1,869	2,450	2,227		0,098	0,100	0,083	0,119
**STO**	1,592	1,349	1,643	1,874	2,240	1,566	2,705	2,038	1,176	2,247	1,857	1,946	1,373	1,543	1,851	2,294		0,088	0,088	0,135
**YAP**	2,697	1,496	2,022	2,175	2,609	1,699	2,916	1,891	1,884	2,358	2,167	2,234	1,983	1,758	2,998	2,252	2,594		0,097	0,109
**RCO**	2,197	1,565	1,869	1,697	2,020	1,705	2,342	2,141	1,394	2,348	1,954	2,136	1,863	2,904	1,926	2,762	2,591	2,333		0,036
**HCM**	1,798	1,305	1,586	1,592	1,582	1,665	1,435	1,307	0,987	1,710	1,483	1,732	1,629	2,129	1,451	1,856	1,607	2,052	6,779	

The lower diagonal area of the table shows the estimated number of migrants per generation (2Nm).

### Genetic Differentiation among Populations

AMOVA based on *F_ST_* (using haplotypes frequencies) and *Φ_ST_* (using genetic distances with Kimura algorithm) were conducted to assess population structure patterns with the ARLEQUIN version 3.5 software [55]. Statistical significance of derived indices was assayed through a nonparametric permutation method (5000 permutations). Likewise, we estimated pair-wise genetic differentiation between populations with *Φ_ST_* and *F_ST_* indices.

### Demographic History and Neutrality Test

We used distributions of the number of pair-wise mutational differences among individuals – or mismatch distributions – to explore demographic patterns of populations with DNAsp version 5.0 software [58]. Raggedness indices were using to analyse goodness of fit of population expansion model according to Harpending [59].

We also test spatial or demographic expansion estimating Tajima’s *D* [60] and Fu’s *F* [61], using DNAsp 5.0. We assessed significance with 1000 permutations.

We used a Bayesian approach implemented in BEAST software (version 1.4.8) [62] to estimate the time of the most recent ancestor (TMRCA) and to investigate patterns of changes in effective population size throughout the coalescent history of this species.

**Table 3:** Estimates of DAMD differentiation (*θ^B^*) through the Bayesian approach implemented in Hickory software including the Deviant Information Criterion (DIC) (95% credibility intervals).

	Full Model		*f = *0 Model		*θ^B^* = 0 Model	
	*θ^B^*	DIC	*θ^B^*	DIC	*θ^B^*	DIC
**Among Populations**	**0.130**	14476.8	0.090	14534.9	0.868	20060.4
	**(0.123–0.140)**		(0.084–0.096)		(0.732–0.988)	
**Among Biogeographic Regions**	**0.037**	3122.6	0.025	3129.1	0.830	3960.1
	**(0.031–0.046)**		(0.020–0.030)		(0.687–0.970)	
**Within Regions**						
**a) Pampeana**	**0.140**	7960.4	0.098	7994.8	0.870	11288.4
	**(0.128–0.152)**		(0.088–0.106)		(0.721–0.991)	
**b) Espinal**	**0.113**	4989.4	0.079	5008.9	0.877	6401.0
	**(0.099–0.127)**		(0.070–0.089)		(0.743–0.988)	
**c) Las Yungas**	**0.027**	1505.7	0.021	1510.9	0.756	1559.2
	**(0.009–0.043)**		(0.010–0.032)		(0.570–0.936)	

Bold type values showed the fittest model.

We examine two different models of rates variation among branches: the strict clock and the uncorrelated lognormal-distributed relaxed molecular clock analyzing ESS values in TRACER [63]. A ESS>200 provided a strong support for a relaxed molecular clock. Under the relaxed molecular clock, we considered three different demographic models (Bayesian skyline, expansion growth and constant size). Analyses using expansion growth and constant size models prior were unsuccessful, failing to reach convergence in multiple analyses.

Mitochondrial rates have been proposed for arthropods to be in the range of 1.5–3.96% pairwise divergence per million years [64]. We apply a mitochondrial mean rate estimate of 2.3% pairwise divergence per million years for our analyses using BEAST version 1.4.8 [62].

Therefore, we selected an uncorrelated lognormal relaxed molecular-clock model using Bayesian skyline tree prior in BEAST with the average number of substitutions per site across the tree averaged to be 0.0115 per million years and unconstrained rates for individual branches.

We use the Bayesian Skyline Plot method (BSP) implemented in the BEAST version 1.4.8 software to visualize the dynamics of the population size fluctuation over time considering GTR substitution model with invariant sites, a constant growth rate for the skyline model and a chain length of 40000000. To test whether our results were influenced by population structure and non-random sampling we conducted extensive experiments using a wide range of different parameter settings. To identify the influence of the number of coalescence groups, analyses were repeated with 4, 6, and 10 groups and the models were compared. Finally, it is assumed that the control region in insects evolves essentially neutrally and that therefore signals of non-neutrality should be associated with population size changes rather than with signals of selection.

Estimates and credible intervals for each parameters and demographic reconstruction were analysed with the software TRACER v.1.1.4 [63] confirming that effective sample size values were better than 200 and that repeated runs with the same settings yielded similar posterior distributions.

**Figure 1:**
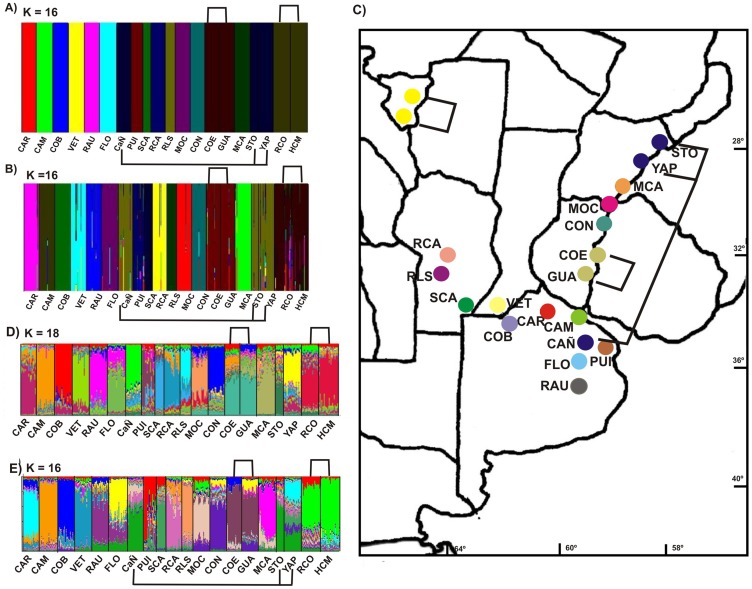
Results of genetic assignment of individuals analysis based on Bayesian method implemented in the programs *BAPS* under mixture of groups of populations model (A), admixture model (B) and spatial model (C) for K = 16, and *STRUCTURE* assuming correlated frequencies and admixed origin of populations for K = 18 (D) and K = 16 (E).

### Gene Flow Pattern and Effective Population Size

Asymmetric migration rates between pairwise studied populations were estimated from haplotypes using Bayesian Markov Chain Monte Carlo (MCMC) coalescent modelling implemented in LAMARC version 2.1.3 [14]. Uniform priors were placed on θ [0.001–1] and M [1–800]. LAMARC analysis consisted of 3 simultaneous searches with automatically adjusted heating temperature using 15 initial chains of 10000 iterations with a burn-in of 1000, followed by 2 final chains of 120000 iterations with a burn-in of 12000. We verified convergence of the likelihood in MCMC chains using TRACER [63].

**Figure 2:**
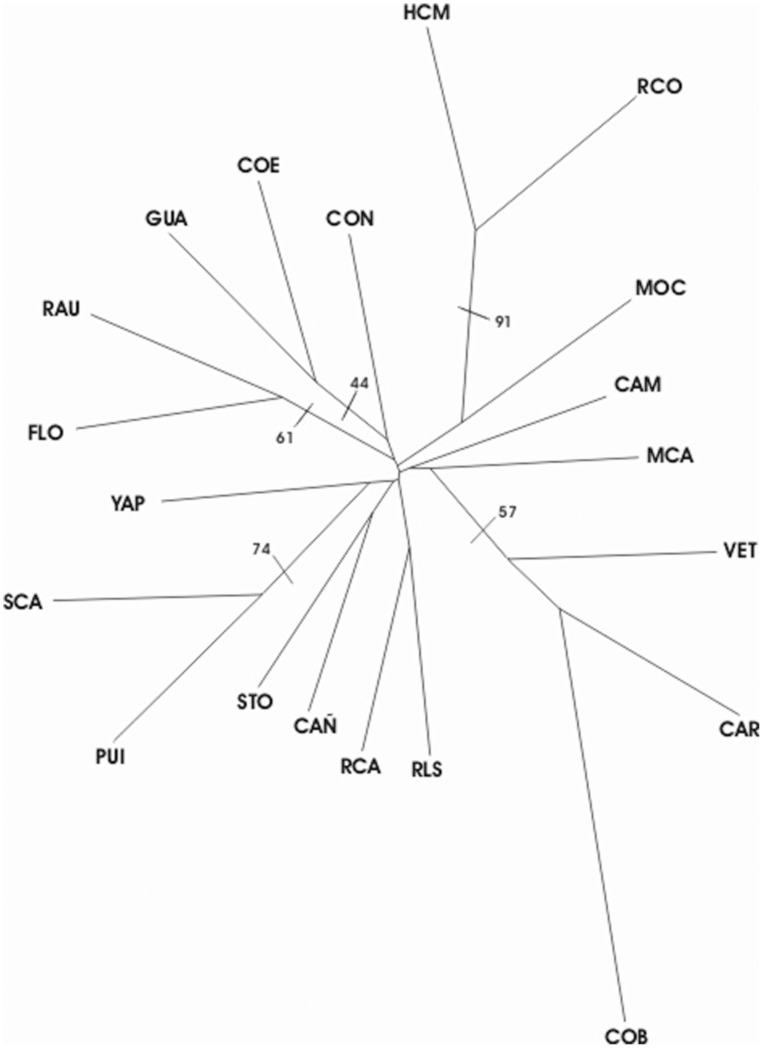
Neighbor- Joining tree of 20 populations of *D. elongatus* based on Nei’s genetic distances. Values of bootstrap support >40 are shown.

## Results

### DAMD-Analysis

#### Intrapopulation genetic diversity

The analysis of DAMD profiles in *D. elongatus* using M13 and INS primers detected a total of 155 informative DNA fragments, 152 of which were polymorphic. No linkage disequilibrium was detected between DAMD markers.

The DAMD variation was quantified (assuming *F_IS_* = 0) in twenty *Argentinean* populations (Table 1). The percentage of polymorphic loci (*PPL*) varied from 100% in RAU, COE, GUA, MCA, STO, YAP, RCO and HCM to 96.8% in SCA, and the mean expected heterozygosity (*H_E_*) ranged from 0.449% in HCM to 0.395% in the sample of SCA. Similarly, YAP (0.601), RAU (0.582) and RCO (0.581) populations had a higher Shannon’s index (*I*), and SCA population (0.488) had a lower Shannon’s index (*I*). The population with the lowest diversity values was SCA, whereas the highest variability was concentrated in populations of Las Yungas and in the north of Espinal biogeographic region.

Spearman partial correlation analysis showed that both *H_E_* and *I* negatively correlated with latitude (r = −0.568, *P* = 0.008; r = −0.374, *P* = 0.01, respectively). Positive correlations were found between *I* and the minimum temperature (r = 0.512, p = 0.02). Whereas *H_E_* was positively correlated with minimum temperature and mean temperature (r = 0.598, *P* = 0.005; r = 0.611, *P* = 0.004).

The results assuming inbreeding and considering the previously *F_IS_* value estimated through isoenzymatic studies (see Materials and Methods) revealed very similar results (data not shown).

**Figure 3:**
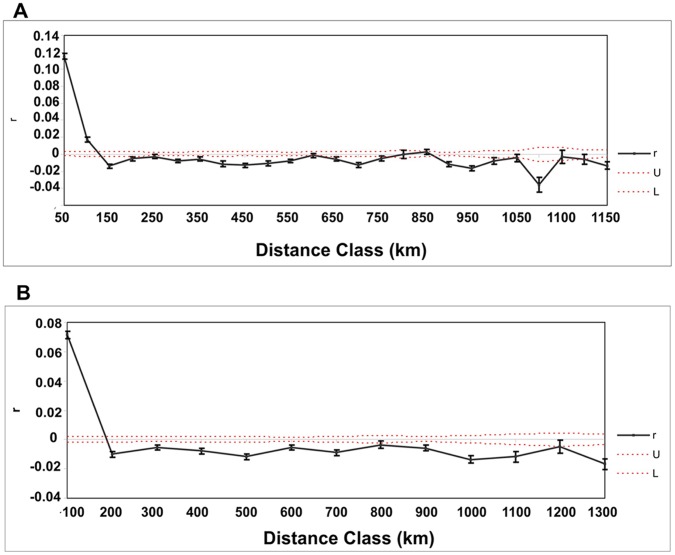
Correlograms showing spatial genetic correlation coefficient (*r*) as a function of geographic distances in the complete study area, considering distance classes of 50 km (A) and 100 km (B). Dashed lines represent upper (U) and lower (L) boundaries of 95% confidence interval under the null hypotheses of no geographic structure. Vertical lines are 95% bootstrapped confidence intervals around each calculated *r* value.

#### Genetic differentiation among populations and gene flow

A hierarchical AMOVA conducted over the 20 populations of the three biogeographic regions indicated that approximately 87% of the DAMD variation arose from genotypic variation within populations (Φ_PT_ = 0,132; P = 0.001), whereas about 12% of the variation was due to differences among populations within regions (Φ_PR_ = 0,124; P = 0.001). A low proportion of the variation (1%) could be attributed to regional differences (Φ_RT_ = 0,010; P = 0.001), which, even though significant, indicated a low structure at the biogeographic level.

When populations are divided according to their distribution across the Paraná River, two groups were obtained: the east Region, including populations located at east of the Paraná River (STO, YAP, MCA, MOC, CON, COE and GUA), and the west Region, containing populations distributed west of Paraná River (RCA, RLS, SCA VET, COB, CAR, CAM, CAÑ, PUI, RAU and FLO). Similar results were obtained when AMOVA was carried out considering east and west regions showing little variation (1%) at the regional level, whereas significant variation was found among (11%) and within (88%) population levels (data not shown).

Pairwise F_ST_ sample comparisons and gene flow estimations (N_e_m) between populations are summarised in Table 2. Although most pairwise gene flow estimates were significant (N_e_m >1), they were insufficient to prevent genetic differentiation.

The highest values of mean gene flow were found in populations located at the northern edge of the studied distribution (RCO, N_e_m = 2.34; YAP, N_e_m = 2.22; MCA, N_e_m = 2.08). The lowest value of mean gene flow was detected for SCA (N_e_m = 1.23). Pairwise comparisons showed the highest N_e_m values among populations of Las Yungas (HCM-RCO, N_e_m = 6.8) and GUA-COE (N_e_m = 3.5) from Pampeana.

In the Bayesian analysis of population differentiation considering all populations, the best model with the lowest DIC value was the full model in which *θ^B^* was equal to 0.13 (Table 3). The DIC value for the *f = *0 model was slightly higher than the DIC value for the full model. Therefore, we could not rule out that some inbreeding occurred in these populations. The *θ^B^* = 0 model showed the highest DIC value, providing strong support for genetic differentiation among the analysed populations.

**Figure 4:**
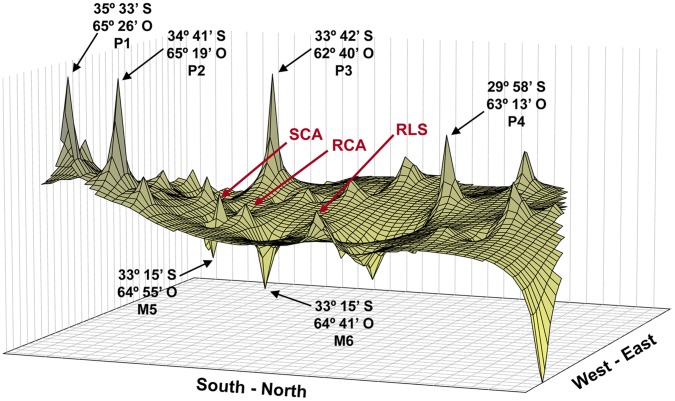
Genetic landscape shape plot showing patterns of spatial genetic distance for 20 populations of *D. elongatu*s. X and Y axes correspond to geographic coordenates and the Z axis (height) corresponds to genetic distance between individuals. Peaks are indicative of areas with high pairwise genetic distances and valleys are indicative of areas of low pairwise genetic distance.

Bayesian approaches were applied to analyse genetic differentiation between regions and among populations within each region (Table 3). The DIC criterion for model choice favoured the full model over all other tested models. The estimates of *θ^B^* among populations within Pampeana, Espinal and Las Yungas biogeographic regions and among biogeographic regions suggest a significant genetic differentiation among populations and low levels of biogeographic structure supporting the AMOVA results.

All three BAPS analyses, i.e., clustering on the individual or population level and using geographic coordinates as informative priors or not, resulted in 16 congruent genetic clusters (Fig. 1 A, B and C). Nonspatial and spatial clustering of groups of individuals resulted in the best partitions with log marginal likelihoods of 37,116.71 and 37,167.75 at P = 1, respectively. In agreement with the studied models, the populations of Las Yungas region (RCO and HCM) fell into a single genetic cluster. GUA and COE from Pampeana region clustered together, and STO and YAP from Espinal biogeographic region and CAÑ from Pampeana fell into a single genetic cluster. In particular, the admixture analysis showed large numbers of individuals with admixed ancestry in the clusters of HCM + RCO and STO+ YAP + CAÑ, indicating that admixed populations are prone to be in the northern distribution of the study area (Fig.1 B).

**Table 4:** Summary of haplotype frequency and genetic diversity indices (gene diversity (h) with their standard deviation and nucleotide diversity (π) with their standard deviation) derivate from the 32 haplotypes found in the 19 populations of *D. elongatus* studied, based on mitochondrial CO1 sequence data.

Haplotype	STO	MCA	YAP	MOC	CON	COE	GUA	CAM	CAR	CAÑ	PUI	FLO	RAU	COB	VET	SCA	RLS	RCA	RCO	Eastern region	Western region
**DE-01**	–	–	–	–	–	–	–	–	–	–	–	–	–	–	–	–	–	–	1	–	–
**DE-02**	–	–	–	–	–	–	–	–	–	–	–	–	1	–	–	3	–	–	–	–	4
**DE-03**	–	–	–	–	–	–	–	–	–	–	–	–	–	1	1	–	–	–	–	–	2
**DE-04**	–	–	–	–	–	–	–	–	1	–	–	–	–	–	1	–	–	–	–	–	2
**DE-05**	–	–	–	–	–	–	–	–	–	–	–	–	–	–	–	2	–	–	–	–	2
**DE-06**	–	–	–	–	–	–	–	–	1	–	–	–	–	–	–	–	–	–	–	–	1
**DE-07**	–	–	1	–	–	–	–	–	–	–	–	–	–	–	–	–	–	–	–	1	–
**DE-08**	–	–	–	–	–	–	1	–	–	1	–	–	–	–	–	–	–	–	–	1	1
**DE-09**	–	–	–	–	–	–	–	–	–	2	–	–	–	–	–	–	–	–	–	–	2
**DE-10**	–	–	–	1	–	–	–	–	–	–	–	–	–	–	–	–	–	–	–	1	–
**DE-11**	–	–	–	1	–	–	–	–	–	–	–	–	–	–	–	–	–	–	–	1	–
**DE-12**	–	–	–	–	–	–	–	1	–	–	1	–	–	1	2	–	–	–	–	–	5
**DE-13**	1	1	2	1	–	–	–	–	–	–	–	–	–	–	–	–	–	–	–	5	–
**DE-14**	–	5	–	1	–	3	2	6	6	7	7	8	7	4	1	1	3	5	–	11	55
**DE-15**	–	–	–	–	–	–	–	–	–	1	–	–	1	–	–	–	–	–	–	–	2
**DE-16**	–	–	–	–	–	–	–	–	–	–	1	–	–	–	–	–	–	–	–	–	1
**DE-17**	–	–	1	–	–	1	–	–	–	–	–	–	–	–	–	–	–	–	–	2	–
**DE-18**	–	–	–	1	–	–	–	–	–	–	–	–	–	–	–	–	–	–	–	1	–
**DE-19**	–	–	–	–	–	–	–	–	1	–	–	–	–	–	–	–	–	–	–	–	1
**DE-20**	–	–	–	–	–	–	–	–	–	–	–	–	–	–	–	–	–	–	2	–	–
**DE-21**	–	–	1	–	–	–	–	–	–	–	–	–	–	–	–	–	–	–	–	1	–
**DE-22**	2	4	2	–	–	–	1	–	–	–	–	–	–	–	–	–	–	–	–	9	–
**DE-23**	–	–	–	–	–	–	–	–	–	–	–	–	–	–	–	–	–	–	7	–	–
**DE-24**	1	–	2	1	12	4	2	2	–	–	–	–	–	–	–	–	1	1	–	22	4
**DE-25**	–	–	–	–	–	2	–	–	–	–	–	–	–	–	–	–	–	–	–	2	–
**DE-26**	–	1	–	5	–	1	2	–	–	–	–	–	–	–	–	–	–	–	–	9	–
**DE-27**	–	–	–	–	–	–	–	–	–	–	2	–	–	–	–	–	–	–	–	–	2
**DE-28**	–	–	–	–	–	–	–	–	1	–	–	–	–	–	–	–	–	–	–	–	1
**DE-29**	–	–	–	–	–	–	–	–	2	–	–	–	–	–	–	–	–	–	–	–	2
**DE-30**	–	–	–	–	–	–	1	–	–	–	–	–	–	–	–	–	–	–	–	1	–
**DE-31**	–	–	–	–	–	–	–	–	–	–	–	–	–	–	–	3	2	–	–	–	5
**DE-32**	–	–	–	–	–	–	–	–	–	–	–	–	–	–	–	–	–	1	–	–	1
**N**	4	11	9	11	12	11	9	9	12	11	11	8	9	6	5	9	6	7	10	67	103
***h***	0.833	0.709	0.917	0.818	0	0.818	0.917	0.556	0.758	0.600	0.600	0	0.629	0.600	0.900	0.806	0.733	0.524	0.551	0.833	0.643
	(0.222)	(0.099)	(0.073)	(0.119)		(0.083)	(0.072)	(0.165)	(0.122)	(0.154)	(0.154)		(0.191)	(0.215)	(0.161)	(0.089)	(0.155)	(0.209)	(0.164)	(0.028)	(0.057)
***π***	0.006	0.006	0.008	0.017	0	0.012	0.017	0.007	0.014	0.002	0.012	0	0.002	0.007	0.011	0.017	0.015	0.008	0.005	0.012	0.009
	(0.004)	(0.004)	(0.005)	(0.009)		(0.007)	(0.009)	(0.004)	(0.008)	(0.001)	(0.007)		(0.001)	(0.005)	(0.008)	(0.009)	(0.009)	(0.005)	(0.003)	(0.006)	(0.005)

The mixture analysis within each biogeographic region supported the global analysis. The highest marginal likelihood was obtained for 10 clusters in Pampeana (20,324.96, 20,296.88) and 6 clusters in Espinal (12,457.04, 12,445.66) with and without geographic information, respectively.

**Table 5:** Pairwise population differentiation estimates of mtDNA *F_ST_* (above diagonal) and pairwise *Φ_ST_* between populations based in mtDNA.

	VET	COB	CAM	CAR	CAÑ	FLO	RAU	PUI	COE	GUA	SCA	YAP	MCA	MOC	CON	STO	RCA	RLS
**VET**		0.029	0.142	0.074	**0.169**	**0.542**	**0.266**	0.132	0.098	0.048	**0.134**	0.091	0.132	**0.132**	**0.716**	0.131	**0.189**	0.097
**COB**	−0.051		−0.074	−0.028	−0.042	0.158	−0.046	−0.070	0.118	0.096	**0.229**	0.226	0.054	**0.229**	**0.795**	**0.300**	−0.071	0
**CAM**	0.163	−0.013		0.011	−0.004	**0.148**	−0.009	−0.022	0.064	0.083	**0.265**	**0.226**	0.089	**0.246**	**0.685**	**0.300**	−0.097	−0.015
**CAR**	**0.142**	0.040	0.078		−0.004	0.184	0.030	0.004	**0.088**	0.062	**0.174**	**0.167**	0.051	**0.175**	**0.621**	**0.215**	−0.007	0.005
**CAÑ**	**0.330**	0.027	0.066	**0.133**		0.130	−0.030	−0.008	**0.142**	**0.112**	**0.248**	**0.249**	0.079	**0.247**	**0.711**	**0.317**	−0.034	0.031
**FLO**	**0.428**	0.086	0.100	0.113	0.059		0.046	0.123	**0.387**	**0.390**	0.528	**0.522**	**0.299**	**0.502**	**1**	**0.719**	0.107	**0.329**
**RAU**	**0.326**	0.014	0.065	0.098	−0.038	−0.015		−0.010	**0.206**	**0.194**	**0.303**	0.333	**0.122**	**0.325**	**0.890**	**0.434**	−0.055	0.078
**PUI**	0.142	−0.004	0.022	0.414	0.066	0.041	0.029		**0.142**	**0.123**	**0.249**	**0.248**	0.079	0.247	**0.711**	0.317	−0.034	0.031
**COE**	**0.269**	**0.197**	0.069	−0.041	**0.287**	0.297	**0.266**	0.104		−0.034	**0.163**	0.047	**0.120**	0.092	**0.371**	0.093	0.095	0.029
**GUA**	**0.241**	**0.188**	0.130	0.004	**0.298**	0.290	−**0.015**	0.046	−0.013		**0.117**	0.010	0.033	−0.003	**0.463**	0.008	0.099	0.025
**SCA**	0.142	0.132	**0.187**	−0.009	**0.286**	0.292	**0.245**	0.062	0.130	0.005		**0.139**	**0.204**	**0.180**	**0.642**	**0.183**	**0.268**	0.031
**YAP**	**0.227**	**0.132**	0.159	0.100	**0.228**	**0.297**	**0.230**	**0.099**	0.015	**0.092**	**0.170**		**0.099**	**0.097**	**0.463**	−0.131	**0.244**	**0.135**
**MCA**	**0.302**	**0.083**	0.043	0.027	**0.124**	**0.113**	**0.090**	−0.016	**0.143**	0.114	0.165	0.064		**0.160**	**0.657**	0.045	0.077	0.068
**MOC**	**0.296**	**0.259**	**0.237**	0.054	**0.363**	**0.355**	**0.333**	**0.101**	0.087	−0.077	0.013	**0.198**	**0.198**		**0.564**	0.137	**0.255**	**0.169**
**CON**	**0.818**	**0.845**	**0.717**	**0.619**	**0.913**	**1**	**0.924**	**0.700**	**0.397**	**0.490**	**0.621**	**0.635**	**0.802**	**0.552**		**0.695**	**0.768**	**0.685**
**STO**	**0.261**	**0.195**	0.011	0.087	**0.394**	**0.593**	**0.396**	0.088	−0.036	0.022	0.141	−**0.162**	0.059	0.149	**0.813**		**0.327**	0.189
**RCA**	**0.198**	0.010	−0.068	−0.031	0.076	0.074	0.040	−0.055	0.022	0.032	0.083	0.014	−0.047	0.130	**0.748**	0.008		−0.011
**RLS**	0.226	0.153	0.099	−0.057	**0.293**	**0.294**	**0.251**	−0.019	−0.026	−0.129	−0.048	0.089	0.074	−0.081	**0.629**	0.035	−0.031	

Comparisons in bold type are statistically significant after Bonferroni correction.

Results from STRUCTURE under the admixture ancestry model with correlated allele frequencies identified 18 genetic groups in the data set. The software identified HCM + RCO and GUA + COE as two genetic clusters (Fig. 1 D). Although the optimum K-value revealed by STRUCTURE was K = 18 with the lower log of marginal likelihood (34,676.54), K = 16 had a near value of log of marginal likelihood (34,702.68), thereby establishing the additional genetic cluster STO+ YAP + CAÑ (Fig. 1 E).

An AMOVA with the genetic groups of populations obtained in the *BAPS* analysis attributed 11% of the overall variance to differentiation among populations (*Φ_PR_* = 0,108; *P* = 0.01) and 89% to variation within populations (*Φ_PT_* = 0,107; *P* = 0.01), whereas the biogeographic regions could not be differentiated. Comparable results were obtained when hierarchical analysis was carried out contemplating eastern and western populations separated by the Parana River (data not shown).

**Figure 5:**
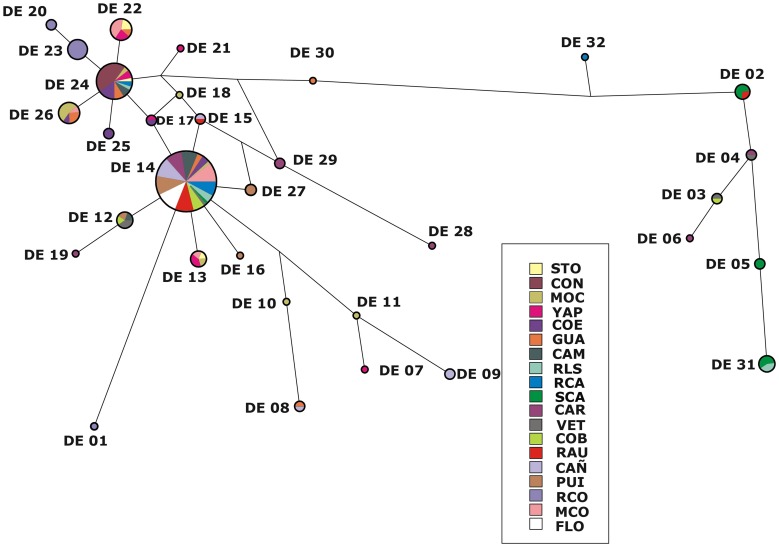
Median-Joining network for the COI mtDNA haplotypes of *D. elongatus*. Inferred median vectors are switched off for clarity. Each circle represents a haplotype, and circle size is proportional to haplotype frequency. Colours indicate the proportion of individuals sampled in different populations within the study area. Branch lengths are proportional to the number of S substitutions per nucleotide site.

#### Phenetic relationships

Neighbour-joining analysis at the population level based on Nei genetic distances (Fig. 2) showed that populations are not grouped according to their biogeographical region, supporting the results of the analysis of population differentiation. The cluster of Las Yungas populations (RCO and HCM) showed the highest bootstrap support (91%). Significant clustering was observed among most populations of Pampeana biogeographic region (except CAÑ and CAM). PUI and SCA are grouped with a support of 74%, RAU and FLO showed a bootstrap of 61%, VET, CAR and COB are grouped with 57% bootstrap support, while COE and GUA presented a bootstrap support of 44%.

**Table 6:** Mismatch distribution raggedness index (r) and estimated demographic parameters Tajima’s *D* and Fu’s *F_S_* based on mitochondrial CO1 sequence data of 19 populations of *D. elongatus* belonging to East and West regions across Parana River.

Population	Tajima’s *D*		Fu’s *F_S_*		Mismatch Distribution	
	*D*	*P*	*F_S_*	*P*	*r*	*P*
**CAM**	−0,359	>0.100	0,101	>0.100	0,185	0,334
**CAR**	−0,393	>0.100	0,263	>0.100	0,194	0,888
**COB**	**−1,477**	0.013	**−1,515**	0.009	0,204	0,552
**VET**	1,642	>0.100	1,642	>0.100	0,230	0,544
**CAÑ**	−0,323	>0.100	0,159	>0.100	0,186	0,678
**PUI**	−1,114	>0.100	−1,049	>0.100	0,195	0,273
**FLO**	–	–	–	–	–	–
**SCA**	−0,959	>0.100	−1,252	>0.100	0,322	0,902
**GUA**	−1,002	>0.100	−1,018	>0.100	0,065	0,103
**COE**	0,238	>0.100	0,406	>0.100	0,076	0,244
**RAU**	**−1,723**	0.028	**−1,863**	0.022	0,313	0,858
**RLS**	1,217	>0.100	1,460	>0.100	0,720	0,976
**RCA**	−1,393	>0.100	−1,500	>0.100	0,503	0,892
**CON**	–	–	–	–	–	–
**MOC**	−0,312	>0.100	−0,478	>0.100	0,084	0,299
**MCA**	0,576	>0.100	−0,083	>0.100	0,327	0,815
**YAP**	−0,765	>0.100	−0,800	>0.100	**0,021**	0,003
**STO**	−0,065	>0.100	−0,065	>0.100	0,306	0,487
**RCO**	−1,636	>0.050	−1,732	>0.100	0,167	0,438
**Eastern region**	**−1,618**	0.033	**−2,083**	0,040	**0,025**	0,040
**Western region**	−0,375	>0.100	−0,427	>0.100	0.069	0,748

The P values for Tajima’s *D*, Fu’s *F_S_* and r indicate the probability that simulated statistic will be more negative than the observed statistic. Bold type values indicate statistical significance (P<0.005).

#### Spatial genetic structure analysis

Mantel tests detected a pattern of isolation by distance (IBD), revealing a significant positive relationship between pairwise genetic and geographical distances for the entire study region (*r* = 0.071, *P* = 0.0009). In Pampeana and Espinal (*r* = 0.136, *P* = 0.0009; *r* = 0.111, *P* = 0.0009, respectively), there was evidence of isolation by distances (IBD). These results suggest that the population of *D. elongatus* in the studied region may have reached equilibrium between genetic drift and migration.

The results of spatial genetic autocorrelation analysis across the entire study region are shown in Figure 3 for distance classes of 50 and 100 km with 95% confidence intervals. The correlogram for the 50-km distance class size showed positive and significant values of *r* at 50 km (*r* = 0.115, *P* = 0.001) and 100 km (*r* = 0.017, *P* = 0.001) with an X - intercept at 127.7 km (Fig. 3 A). The correlogram for the 100-km distance class size detected positive and significant values of *r* at 100 km (*r* = 0.071, *P* = 0.001), but significantly negative values for distances up to 200 km, with an X- intercept at 187.7 km (Fig. 3 B). These results suggest that populations of *D. elongatus* located at shorter distances than 100 km are more genetically similar to each other than expected by an IBD pattern and that the genetic differentiation for populations located at distances over 200 km is higher than expected from random spatial distribution.

The genetic landscape shape analysis showed a strong pattern of higher genetic differentiation among populations in the central area of the distribution range (Fig. 4). The highest peaks are located in the central-west edge of the studied area corresponding to south-east of Córdoba Province and south-west of Santa Fe Province between SCA and VET populations (Fig. 4, P3). Two peaks occurred at the south-western edge of Córdoba Province (Fig. 4, P1 and P2), and a 4^th^ peak was observed at the north-eastern edge of Córdoba Province (Fig. 4, P4). The landscape plot showed low genetic distances between individuals (represented as a trough) in the western limit of the Espinal region, M5 and M6, indicating low barriers to gene flow.

**Figure 6:**
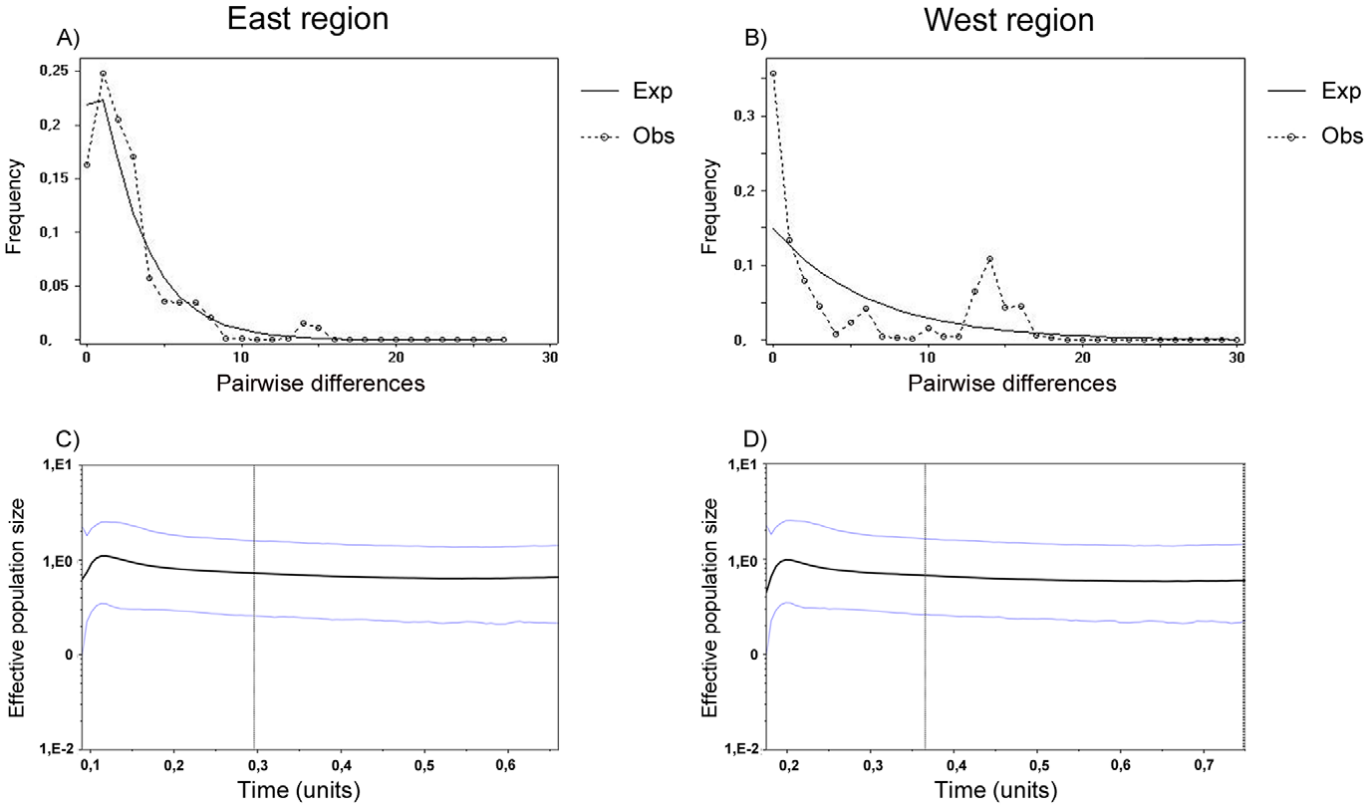
Mismatch distributions (above) and Bayesian Skyline Plots (below) depicting the demographic history for each region across Parana River of *D. elongatus* using CO1 gene sequence. For the mismatch distributions, the solid lines show observed frequency distribution while the dashes lines show the distribution expected under the sudden-expansion model. For the skyline plots, the *x* axis is time of mutations per site before present; the *y* axis is the expressed population size estimated in units of Ne*μ* (Ne: effective population size, *μ*: mutation rate per haplotype per generation). The central line represents the median value for the log10 of the population size and the dashed lines represent the upper and lower 95% credible intervals.

### Mitochondrial DNA Analysis

#### Intrapopulation genetic diversity

A region of 527 bases of the mtDNA cytochrome oxidase I (COI) was sequenced in each of 170 individuals collected from 19 populations in three biogeographic regions, resulting in 39 polymorphic sites and 32 different haplotypes (Table 4, see Appendix S1). All differences between haplotypes were due to substitutions (31 transitions and 10 transversions).

The most common and widespread haplotype (DE-14) consisted of 66 individuals and was detected in 15 out of 19 analysed populations (MCA, MOC, COE, GUA, CAM, CAR, CAÑ, PUI, FLO, RAU, COB, VET, SCA, RLS and RCA). The second most frequently observed variant (DE-24) was found in 26 individuals appearing in 9 populations (STO, YAP, MOC, CON, COE, GUA, CAM, RLS and RCA) (Table 4).

**Table 7:** Time of divergence among *D. elongatus* populations based on Bayesian coalescent estimation.

Population	T_MRCA_
CAM	**0.241** (9,2 E^−2^–0.419)
CAÑ	**0.274** (0.101–0.487)
CAR	**0.730** (0.354–1.174)
COB	**0.720** (0.352–1.174)
VET	**0.725** (0.353–1.174)
PUI	**0.190** (5.35 E^−2^–0.363)
FLO	**0.171** (4.09 E^−2^–0.330)
RAU	**0.720** (0.353–1.174)
GUA	**0.662** (0.296–1.081)
COE	**0.238** (9.24 E^−2^–0.418)
CON	**9.33** E^−2^ (2.33 E^−2^–0.176)
MOC	**0.286** (0.111–0.500)
MCA	**0.241** (9.24 E^−2^–0.419)
YAP	**0.285** (0.109–0.498)
STO	**0.232** (8.43 E^−2^–0.413)
SCA	**0.739** (0.708–1.174)
RCA	**0.739** (0.354–1.174)
RLS	**0.739** (0.353–1.174)
RCO	**0.242** (8.71 E^−2^–0.435)

Time of divergence among populations are in million years. Bayesian coalescent estimation of time to most recent common ancestor (TMRCA) with 95% highest posterior density modeled assuming a relaxed molecular clock as implemented in BEAST are shown.

The Pampeana and Espinal biogeographic regions shared 6 haplotypes, whereas no haplotyes were shared between Las Yungas and the remaining biogeographic regions. In Pampeana, 14 private haplotypes were identified, 5 of which occurred in a single individual (singletons). In Espinal, 10 private haplotypes were detected, 6 of which were singletons. Populations with a higher degree of private haplotypes were CAR (four private haplotypes) and MOC and RCO (both with three private haplotypes). When populations were grouped according to their geographic position with respect to Paraná River, we demonstrated that the east and west regions only shared 3 haplotypes. The most frequent haplotypes (DE-14 and DE-24) and DE-08 were present in GUA and CAÑ. In the east region, 10 private haplotypes were detected, 5 of which occurred in a single individual, whereas in the west region, 15 private haplotypes were detected, 5 of which were singletons.

Genetic diversity indices are shown in Table 4. Haplotype (*h*) and nucleotide diversity (*π*) ranged from 0 to 0.917 and 0 to 0.017, respectively. YAP and GUA showed the highest haplotype diversity, whereas CON and FLO showed the lowest values of haplotype diversity. The highest values of nucleotide diversity were detected in MOC, GUA and SCA, and CON and FLO showed the lowest values (Table 5).

Haplotype diversity was found to be positively and significantly correlated with maximum annual temperature (*r* = 0.466; *P* = 0.044).

**Figure 7:**
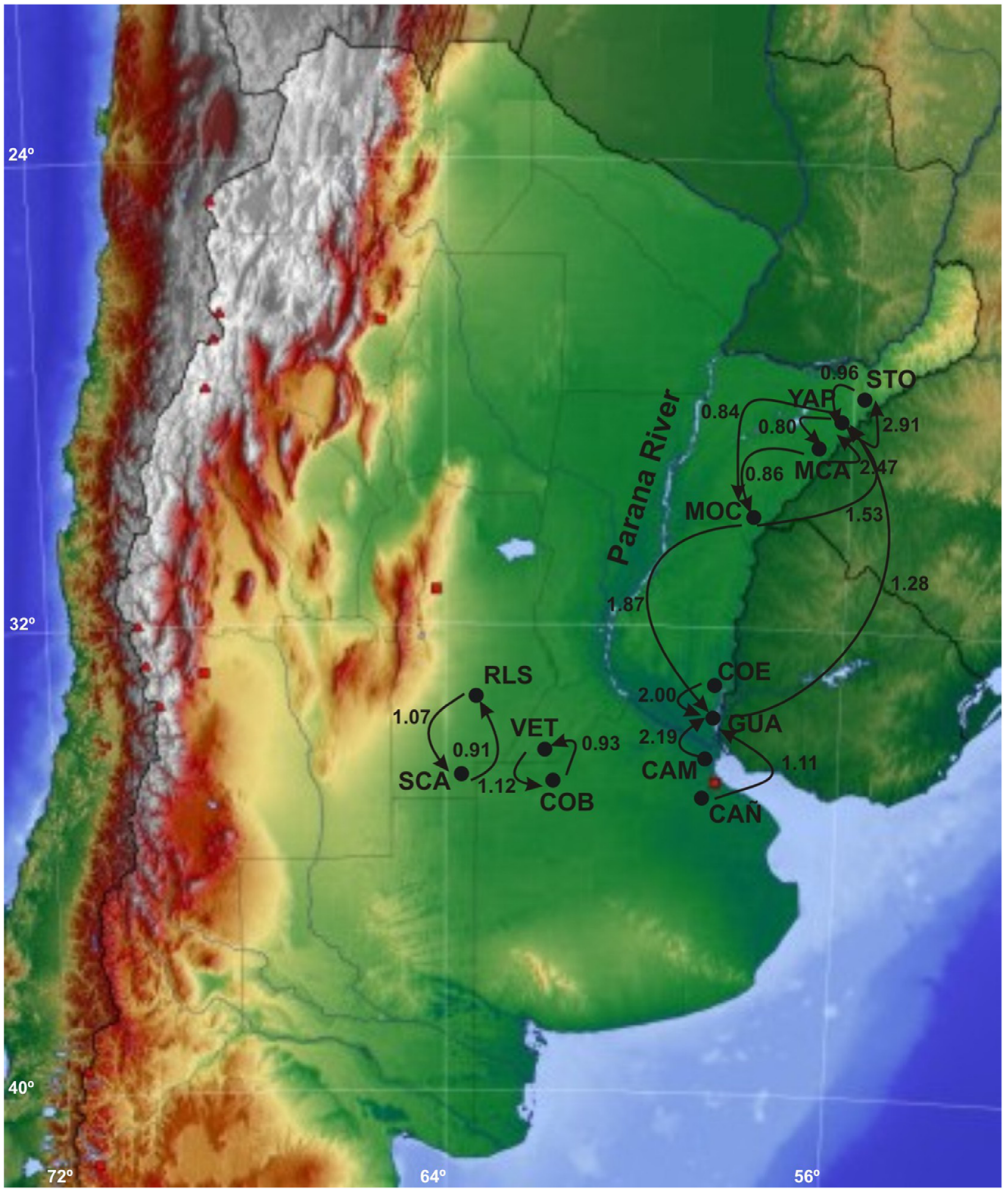
Bayesian estimates of historical asymmetrical migration between populations of *D. elongatus*, based on the program LAMARC. Arrows indicate the direction of migration rates which values are over 0.8.

#### Phylogenetic relationships among haplotypes

The COI median joining network displayed a genealogy with two main haplotypes separated by two mutational steps (Fig. 5). The haplotype network suggested that the most common haplotypes (DE-14 and DE-24) might be proposed as ancestral haplotypes by their internal position in the network, the number of lineages that arise from them, and their frequencies.

Light haplotype structuring was evident because of the fact that some star-like phylogenies were found in different parts of the network. Some of the star-like groups were found in a particular region or in adjacent populations with related haplotypes radiating from a single common haplotype. This scenario was observed for the cluster around DE- 24, which was found only in the eastern region of the sampled distribution. Three haplotypes (DE-13, 22 and 26) were exclusively from the east edge of the studied area (populations between STO and GUA), whereas haplotype DE-12 was distributed exclusively from VET to FLO (southern region of the distribution).

#### Genetic differentiation among populations

The AMOVA that accounted for frequencies and divergence between haplotypes revealed that only 5% of the genetic variance was found among biogeographic regions *(Φ_CT_* = 0.202, *P* = 0.07), differences among populations within regions accounted for 19% (*Φ_SC_* = 0.201, *P* = 0.0001) of the total variation, while 76% of the variance could be attributed to within-population variability (*Φ_ST_* = 0.242, *P* = 0.0001).

The parallel analysis using F_ST_ statistics showed a higher level of variation among biogeographic regions (6.7%) (*F_CT_* = 0.067, *P* = 0.028) and lower levels of differentiation among populations within biogeographic regions (18.5%) and within populations (74.7%) (*F_SC_* = 0.198, *P* = 0.0001 and *F_ST_* = 0.252, *P* = 0.0001, respectively). The F_ST_ statistics values were generally lower, suggesting the importance of haplotype divergence in the population structure pattern.

Differences were also found when considering east and west regions separated by Paraná River. A higher level of genetic differentiation was evident among regions when the geographic groups were taken into account for the AMOVA analysis. Differences among east and west regions accounted for 15.8% (*F_CT_* = 0.151, *P* = 0.0001) of the total variation, while only 12.7% (*F_SC_* = 0.281, *P* = 0.0001) of the variance could be attributed to variation among populations within regions and 71.5% could be attributed to within-population variability (*F_ST_* = 0.158, *P* = 0.0001). The same pattern was found for the analysis using *Φ_ST_* statistics. Differences among regions accounted for the 11.8% (*Φ_CT = _*0.168, *P* = 0.004) of the variance, while 14.8% of the variation was attributed to differences among populations within regions (*Φ_SC_* = 0.266, *P* = 0.0001) and 73.4% to differences within populations (*Φ_ST_* = 0.118, *P* = 0.0001).

Pairwise comparison *Φ_ST_* test among the 19 populations demonstrated significant differences in 66 of 153 comparisons after sequential Bonferroni correction (Table 5). Pairwise *Φ_ST_* values ranged from −0.004 to 1. CON was found to be exceptionally highly differentiated from every other population, while RLS, RCA and CAR showed the lowest values of differentiation.

Pairwise F_ST_ varied from −0.004 to 1, and significant values were detected in 75 of the 153 comparisons (Table 5). The highest values were found in pairwise comparisons including CON population, while the lowest values were detected in RCA and COB.

#### Demographic history and neutrality test

Results of Tajima’s D, Fu’s Fs tests and raggedness statistic of mismatch distribution analysis are provided in Table 6.

Tajima’s D and Fu’s Fs tests were only significant for the east region (populations located at east of Paraná River). These results were confirmed by the observed mismatch distributions, which closely matched those expected under a model of sudden expansion. The mismatch distribution plot for the east region was smooth and unimodal, indicating a population expansion, whereas the multimodal pattern of the west region mismatch distributions may suggest strong population subdivision and a stable population size (Table 6, Fig. 6 A and B).

At the population level, COB and RAU have negative and significant Tajima’s D (*P* = 0.013 and 0.028 respectively) and Fu’s Fs values (*P* = 0.009 and 0.022, respectively). The mismatch distribution for YAP was clearly unimodal and closely matched the expected distributions under the expansion model, whereas COB and RAU showed bimodal distributions, suggesting that expansion events were not detected with this analysis (data not shown).

Historical demographic reconstructions (BSPs) of the east and west regions are shown in Fig. 6. After a long phase of demographic stability, the east region appeared to experience an accelerated demographic expansion phase approximately 150000 years before present, followed by an abrupt decrease in population size approximately 100000–75000 years ago (Fig. 6 C). A somewhat similar trend was observed in the west region for which a slower population growth would have occurred at around 250000 years ago after a relatively constant population size over time and a strong decline nearly 150000 years before present (Fig. 6 D).

The estimated T_MRCA_ for the east region was approximately 0.66 Myr (660000 years) in the late Pleistocene glacial cycles (mean = 0.662, 95% CI = 0.296–1.081, ESS = 1224.3). The mean Bayesian T_MRCA_ for the west region was 0.74 Myr (740000 years) (mean = 0.748, 95% CI = 0.366–1.194, ESS = 1043.2). All sampled populations coalesce at approximately 740000 years, indicating that the west region is the most ancestral.

Individual population sizes were traced back to the origin of each population as inferred by BSP analysis. YAP Bayesian skyline plot showed a population size growth event at approximately 260.000 years ago, followed by a relatively stable population size (data not shown).

T_MRCA_ values for each population dated back to the late Pleistocene, with mean values between 0.09–0.739 Myr (Table 7). CON had the highest value of differentiation and presented the lowest value of T_MRCA_ (mean = 9.33 E^-2^, CI = 2.33 E^-2^–0.176), suggesting a very recent colonisation. GUA showed a similar T_MRCA_ (mean = 0.662, CI = 0.296–1.081) to the entire east region, indicating that this population is the most ancestral of the eastern region.

#### Historical population size and migration rates

The results obtained from the *LAMARC* analysis showed low values of historical gene flow, ranging from 10^−5^ to 2.91. The coalescent model revealed asymmetrical migration rates from southwest to northeast. The highest migration rates were detected among nearby populations located in the east region (Fig. 7), with the exception of VET-COB and RLS-SCA, which belong to the west region. CON is effectively isolated with little gene flow (0.0003<Nem<0.311), even from neighbouring populations. It also provides an extremely low level of migrants to other east region populations.

Our results showed high levels of migration from CAM and CAÑ (the nearest populations to the Paraná River in the west region) to the eastern populations, but no migration in the reverse direction, indicating a possible colonisation route for this species (Fig. 7).

## Discussion

*Dichroplus elongatus*, a widely distributed South American grasshopper, is considered to be an agricultural pest of crops and forage grasses of great economic importance [19], [20]. We examined the genetic variability using nuclear and mitochondrial markers in an attempt to characterise the population structure of this species of grasshopper. This analysis of 20 Argentinean populations of *D. elongatus* using Direct Amplification of Minisatellite Regions (DAMD) and a 527 bp fragment of COI mtDNA sequences demonstrated higher levels of intraspecific variation comparable with other orthoptera taxa [65], [66], [67].

DAMD analysis in *D. elongatus* showed that genetic variability was characterised by a decrease in *PLP*, *H_E_* and *I* as a function of latitude and temperature. The low level of DAMD variability detected in southern populations could be related to the fragility of the populations due to the extension of agroecosystems in the southernmost studied area. Populations at high latitude in the studied area include the southernmost analysed populations of Pampeana region and the westernmost studied populations of Espinal. All of the studied high-latitude populations belong to geographic provinces that are subject to intense agricultural exploitation (Córdoba, Buenos Aires and Santa Fe). Grasshopper control through agropesticides may explain the reduction in population size and the consequent loss of genetic diversity in these populations.

The DAMD population structure estimated by AMOVA showed that the distribution of the variation is not associated with the biogeographic or geographic region of origin and that the main genetic differentiation lies within populations. Accordingly, the Bayesian analyses of genetic differentiation showed that the “full model” better explains the detected variation, indicating genetic differentiation among populations and some local inbreeding. However, the model that implies differentiation between populations and lack of local inbreeding also managed a good fit, which suggests that local inbreeding is low.

The analysis of DAMD population structure using Bayesian cluster approaches through *BAPS* and *STRUCTURE* software efficiently identified the number of clusters or ideal populations. *BAPS* demonstrated the existence of at least 16 ideal populations, i.e., genetically differentiated clusters. The software recognises sampled populations in Las Yungas (RCO and HCM) and two populations of Pampeana (COE and GUA) as an ideal population and identifies a cluster consisting of two nearby populations of Espinal and a distant Pampeana population (STO, YAP, CAÑ). *STRUCTURE* software identified 18 ideal populations suggesting two clusters, one of which brings together RCO-HCM and another cluster grouping COE-GUA. The a posteriori probability for K = 16 introduced only minor differences and identified an additional cluster formed by STO, YAP and CAÑ.

This result confirms the level of DAMD differentiation between populations detected by AMOVA, Bayesian analysis of differentiation and the phenogram, suggesting a significant constraint on gene flow, even among nearby populations, or a low level of shared polymorphisms. The analysis of the assignment of individuals through *BAPS* considering the option of “admixture of genomes” gives additional information. HCM, RCO, STO and YAP populations contain more individuals with a mixture of ancestries suggesting the importance of gene flow in the observed differentiation. Estimates of gene flow between pairs of populations support these results, as they showed increased gene flow between populations grouped by the probabilistic assignment of individuals (HCM-RCO) (COE-GUA).

The strong signal of detected population structure is not common in species of Orthoptera. A microsatellite loci analysis of 24 populations of *Mioscirtus peccary* in the Iberian Peninsula identified only 8 clusters [66]. Similar results were detected by analysing the variability in microsatellite loci of *Locusta migratoria*. Analysis of 24 populations in Europe, China and Madagascar identified 7 ideal populations [67]. In both species, an important population structure was detected, but it was less than that demonstrated for *D. elongatus* in Argentina.

The spatial genetic structure analysis not only demonstrated that *D. elongatus* was genetically highly diverse, but also that the DAMD variation among populations was geographically associated. The autocorrelation results are in agreement with the Mantel test results. Both approaches demonstrated a significant increase in genotypic distances among populations with growing geographic distance. Analysis of variation in DAMD loci on a large scale by use of Mantel test showed IBD when considering the entire studied area, or the separate biogeographic regions of Pampeana and Espinal (data not shown). Genetic differentiation tends to increase with increased geographic distance, but that relationship explains only 12% of the variation. The remaining variation is due to variability within populations. Spatial autocorrelation analysis showed a high correlation among individuals at the smallest distances. We have detected a consistent pattern of fine-scale genetic structure with positive autocorrelation for populations separated by less than 100 km, whereas populations separated by more than 200 km showed greater genetic differences than expected from a random distribution. The autocorrelation and Mantel test results indicate that proximate populations are genetically more alike than more distant ones.

Positive autocorrelation signals may also be due to spatial patterns shaped by natural selection, but only at selected or linked loci [68]. This scenario is not applicable to the *D. elongatus* DAMD loci. Our results suggest that although migration and genetic drift occur among adjacent populations, measurable relatedness among neighbouring populations declines with distance, and dispersal over distances greater than 200 km is not typical, whereas effective gene flow may occur for populations separated by less than 100 km. Thus, besides local endogamy, restrictions in gene flow in *D. elongatus* may lead to a non-homogenous spatial structure and justify the variation through space. In general, the DAMD pairwise gene flow estimates of neighbour populations are relatively low (1<N_e_.m<2), supporting the importance of restrictions in gene flow in the signals of autocorrelations.

The environmental characteristics typifying the different habitats that shape the conditions of a landscape can either limit or promote the movement of individuals in populations and so the extent of genetic connectivity [69]. Among physical barriers that may reduce genetic connectivity (such as rivers or mountains), anthropogenic features have been shown to slow animal dispersal. In *D. elongates,* the genetic landscape shape plot shows a strong restriction in gene flow between populations from regions that would have been more disturbed by anthropogenic activities (SCA, RLS, RCA and VET populations). Road edges and grassy margins are common features in most agricultural landscapes [70]. These elements may act as corridors for dispersal and facilitate the exchange of individuals among more distant patches. Grassy margins may also provide temporal retreats from which individuals could recolonize grassland patches after disturbances [71]. Córdoba, Buenos Aires and Santa Fe Provinces correspond to highly exploit agricultural areas of Argentina, and fields in this region lack of grassy margins. Such agroecosystem could explain the genetic discontinuities found nearby.

The analysis of mtDNA sequence of *D. elongatus* identified 32 haplotypes and demonstrated large values of nucleotide (0.002–0.017) and haplotype (0.524–0.917) diversity, but no geographic pattern was detected. Most populations share one or both of the most frequent haplotypes (DE-14 and DE-24). The presence of shared haplotypes in most populations may be explained by one of two scenarios: restricted gene flow among local populations or the predominance of ancestral haplotypes [72].

The limited gene flow among populations indicated by minisatellite analysis and *LAMARC* studies support the second scenario. However, the magnitude of estimated gene flow between pairwise populations according to mtDNA data was different from some of the estimates based on F_ST_ values using DAMD data. The results from *LAMARC* analysis demonstrated that there was some directional bias in dispersal, especially between local populations of *D. elongatus* in different geographic regions. mtDNA (reflecting female movement) suggested a marked directionality of gene flow from western to eastern populations and at a minor level from south to north, suggesting a directional movement of females towards the north-eastern populations.

The relationship between haplotype and nucleotide diversity may be explained by different demographic frameworks [73]. Advanced studies using coalescent-based approaches allow for an effective detection of historical demographic changes [12], [13], [14].

When values of haplotype (*h*>0.5) and nucleotide (*π*>0.5%) diversity are high, the population is stable with a relatively long evolutionary history. Populations of STO, MCA, MOC, COE, GUA, CAR, PUI, VET, SCA and RLS meet this condition, suggesting apparent population stability.

Low values of *π* and high values of *h* suggest the existence of small populations that have suffered recent population growth. This situation is observed for RAU, where haplotype diversity is relatively high and nucleotide diversity is relatively low. This population has a relatively high frequency of one of the ancestral haplotypes and no private haplotype. Neutrality tests, conducted through the Tajima’s *D* and Fús *Fs* indices, support the hypotheses of a recent expansion in RAU from a relatively small population.

When *π* values are high and *h* values low, the population may have high genetic differentiation. This situation is observed for CAM and RCA, which have the lowest values of *h* (with the exception of FLO and CON) and high nucleotide diversity. These results may reflect the divergence level of both populations.

When both *π* and *h* values are low, the population might have undergone a reduction in population size or a recent colonisation event that generated few mitochondrial lineages. CON, FLO and RCO are characterised by low values in both indices, reflecting that these populations may have experienced a reduction in population size in the past. The estimated T_MRCA_ showed that CON and FLO are the youngest populations (90000 and 170000 years before present supporting the hypothesis of a very recent colonisation of these sites, whereas RCO is an older population (242000 years before present), suggesting that low genetic diversity may reflect anthropogenic pressure.

The mtDNA diversity analysis reveals that *D. elongatus* exhibited a higher degree of variation with respect to other grasshopper species. In *Mioscirtus wagneri,* the analysis of 16S and COII partial sequences in 24 natural populations of Iberian Peninsula showed values of haplotype (0–0.68) and nucleotide diversity (0–0.0006) that were substantially lower than those observed in the present study [65]. Genetic analysis of mtDNA COI partial sequences among 12 populations of the rice grasshopper *Oxya hyla intricate*, which is a rice pest in southeast Asia, showed moderate values of genetic diversity (*h* = 0.362–0.80; *π* = 0.0006–0.0026) [67].

AMOVA results based on *Φ_ST_* and *F_ST_* statistics showed some differences. The AMOVA based on *Φ_ST_* revealed greater differentiation among populations (14.8%) than the AMOVA based on haplotype frequencies (*F_ST_*) (12.7%), indicating the relative importance of the divergence between haplotypes in the pattern of observed population differentiation. AMOVA analysis also indicated that ∼70% of the variation could be explained by differences within populations, while only ∼15% of the variation was attributed to differences among populations within regions. A large component of the variation (∼12%) was observed among geographic regions (east and west sides of Paraná River). Pairwise comparisons based on the frequency of haplotypes and the distances between them (*Φ_ST_*) indicated that CON is the most differentiated population from the others. The high genetic differentiation detected between populations located on both margins of the Paraná River suggests significant restrictions in gene flow mediated by females and substantial large scale geographical differentiation.

Phylogenetic inference based on the haplotype network displayed a “star-like” shape with DE-24 being the most probable ancestral haplotype. This structure reveals a shallow geographic structure.

The present results are indicative of significant genetic differentiation of mtDNA lineages among the eastern and western populations, although these regions do not appear to display a strong phylogeographic signal.

Different populations exhibit different histories, and even it is wrong to assume that ancestral or refugial populations have stable population histories [74].

Pleistocene era was characterised by cyclic climatic oscillations between warm and cold periods affecting population isolation in many species through recurrent glaciations [75], [76].

The analysis based on the molecular clock model for COI haplotypes supports the hypothesis that the *D. elongatus* populations from the studied area of Argentina originated 740000 years ago during Middle Pleistocene (781000–126000 years before pesent). Moreover, our T_MRCA_ analysis indicates that populations located west of Paraná River (an important geographical barrier in the studied area) are the most ancient; however, populations located at east of Paraná River showed higher values of genetic diversity, suggesting secondary contact.

Paleo-taxonomic approaches suggested that environmental conditions should be some different in west and east regions of Paraná River during Pleistocene. The west region must have been more cold and arid environmental conditions, with a manifest predominance of open environments whereas west region of Paraná River must have been associated to more benign conditions, i.e., warm and humid [77]. During the Pleistocene, the Paraná River would have experimented oscillations in water level and would have left act as a geographical barrier [77], [78]. This scenario would allow the entry of west grasshoppers to warm and humid areas in southwest-northeast direction as detected by the *LAMARC* analysis in Middle Pleistocene. Further oscillations in water levels of the Paraná River during Upper-Middle Pleistocene transition, may also explain a secondary contact among both regions across the River in west-east direction. In agreement, the pairwise mismatch analysis in eastern region fitted into a pattern of population undergoing expansion, supporting an extremely dynamic history of eastern populations.

The migration rate analysis showed that one of the most likely routes of colonisation of the eastern region was through the area containing the CAM and CAÑ populations, which was formed by a slightly elevated block during Middle Pleistocene [79]. Our analysis demonstrates that from CAM and CAÑ, western individuals dispersed to GUA and from there colonised the eastern region. This finding is supported by the analysis of the time of the most recent common ancestor (T_MRCA_), which points to GUA as the most ancient population of the eastern region.

According to the BSP, after a phase of constant population size, the eastern and western regions appear to have experienced a demographic expansion, beginning approximately 150000 and 250000 years before present, respectively. Paleoenvironmental studies of the landscape in these regions during Late Pleistocene, but before Last Glacial Maximum (LGM), suggest the existence of suitable environmental conditions characterised by a slight humidity elevation and grassland vegetation [77], supporting the results of BSP.

Even though, Pleistocene climatic changes affect patterns of intraspecific diversity [80], recent approaches have demonstrated that other causes, such as behavioural and morphological adaptations, may also influence dispersal and differentiation [81], [82]. Competition among males for local resources or mating chances (intrasexual selection) envisages a male-biased dispersal in polygynous species. Sex biases in dispersion may also generate structured populations affecting selective processes in local populations with different adaptive conditions [83].

The use of biparental (DAMD) and uniparental (mtDNA) inheritance markers in *D. elongatus* has provided answers to multiple questions about the population structure and dispersal abilities of these species. There are differences among AMOVAs based on minisatellite loci and AMOVAs based on mtDNA sequences, showing male-biased gene flow. Additionally, different populations of *D. elongatus* have reproductive and morphological differences, including male sexual selection favouring larger individuals and phenotypic variation as a result of local adaptation to season length and number of generations along a latitudinal gradient [22], [84]. These selective differences may promote the dispersion of males to more favourable regions contributing to shape the observed scenario.

Our results show that the complex demographic history, effective migration, contemporary anthropogenic pressures and landscape heterogeneity could determine the genetic structure among populations of *D elongatus*. Although climatic events of the Pleistocene have had a marked effect on the historical distribution and intra-specific divergence of *D. elongatus*, the extreme human land use and recent insecticide approaches used to control pest expansion could affect the intraspecific variation of this species. The dispersing males of *D. elongatus* may contribute to diminish the chances of genetic loss associated with recent anthropogenic activities in the extended Argentinean agro ecosystem. However in *D.elongatus* the male biased dispersal may be insufficient to prevent some level on local inbreeding detected through nuclear markers. Further studies examining genetic population structure over time and space are essential to analyse the effect of programmes for combating this grasshopper species.

## Supporting Information

Appendix S1 **Variable sites defining the 32 haplotypes recovered from the 19 populations of D. elongatus studied.**
(DOC)
