# Supplementary material for: Spatial Genetic Structure and Mitochondrial DNA Phylogeography of Argentinean Populations of the Grasshopper Dichroplus elongatus
Source: PLoS One. 2012 Jul 30;7(7):e40807. doi: 10.1371/journal.pone.0040807 (PMC3408447; doi:10.1371/journal.pone.0040807)
Supplement: Supplementary file 1 [file pone.0040807.s001.doc]

	9	14	17	26	29	39	47	53	71	100	101	104	126	128	134	143	184	194	233	281	282	293	303	324	335	371	374	382	387	389	409	410	419	434	449	461	464	497	503
**DE-01**	C	G	A	A	C	A	T	T	A	G	T	T	A	C	A	T	C	C	T	C	T	A	C	C	T	A	A	C	T	G	T	C	C	T	T	C	A	A	C
**DE-02**	T	A	C	G	.	.	A	C	C	.	.	C	G	T	.	.	T	T	A	T	.	.	.	T	.	.	.	.	.	A	.	T	.	.	.	.	.	.	.
**DE-03**	T	A	C	G	T	.	A	C	C	.	.	C	G	T	.	.	T	T	A	T	.	.	.	T	.	.	.	.	.	A	.	T	.	.	A	.	.	.	.
**DE-04**	T	A	C	G	T	.	A	C	C	.	.	C	G	T	.	.	T	T	A	T	.	.	.	T	.	.	.	.	.	A	.	T	.	.	.	.	.	.	.
**DE-05**	T	A	C	G	T	.	A	C	C	.	.	C	G	T	.	.	T	T	A	T	.	.	.	T	.	.	.	.	.	A	.	T	.	.	.	T	.	.	T
**DE-06**	T	A	C	G	T	.	A	C	C	.	.	C	G	T	.	.	T	T	A	T	.	C	.	T	.	.	.	.	.	A	.	T	.	.	A	.	.	.	.
**DE-07**	T	.	.	.	.	.	.	C	.	.	.	C	G	.	.	C	T	.	.	.	.	.	.	.	.	G	.	.	.	.	.	.	T	.	G	.	.	.	.
**DE-08**	T	.	.	.	.	.	.	C	.	.	.	C	G	.	.	.	T	.	.	.	C	.	.	.	.	.	G	.	.	A	.	.	.	.	G	.	.	.	.
**DE-09**	T	.	.	.	.	.	.	C	.	.	.	C	G	.	.	.	T	.	.	.	C	.	.	.	.	G	.	.	.	.	.	.	T	.	G	.	G	.	.
**DE-10**	T	.	.	.	.	.	.	C	.	.	.	C	G	.	.	.	T	.	.	.	.	.	.	.	.	.	.	.	.	A	.	.	.	.	G	.	.	.	.
**DE-11**	T	.	.	.	.	.	.	C	.	.	.	C	G	.	.	.	T	.	.	.	.	.	.	.	.	G	.	.	.	.	.	.	T	.	G	.	.	.	.
**DE-12**	T	.	.	.	.	.	.	C	.	.	.	.	G	.	.	.	T	.	.	.	C	.	.	.	.	.	.	.	.	.	.	.	.	.	.	.	.	.	.
**DE-13**	T	.	.	.	.	.	.	C	.	.	.	.	G	.	.	.	T	.	.	.	.	.	.	.	C	.	.	.	.	.	.	.	.	.	.	.	.	.	.
**DE-14**	T	.	.	.	.	.	.	C	.	.	.	.	G	.	.	.	T	.	.	.	.	.	.	.	.	.	.	.	.	.	.	.	.	.	.	.	.	.	.
**DE-15**	T	.	.	.	.	.	.	C	.	.	.	.	G	.	.	.	T	.	.	.	.	.	.	T	.	.	.	.	.	.	.	.	.	.	.	.	.	.	.
**DE-16**	T	.	.	.	.	.	.	C	.	.	.	.	G	.	.	.	T	.	.	.	.	.	T	.	.	.	.	.	.	.	.	.	.	.	.	.	.	.	.
**DE-17**	T	.	.	.	.	.	.	C	.	.	.	.	G	.	.	.	T	.	.	T	.	.	.	.	.	.	.	.	.	.	.	.	.	.	.	.	.	.	.
**DE-18**	T	.	.	.	.	.	.	C	.	.	.	.	G	.	.	.	T	.	.	T	.	.	.	T	.	.	.	.	.	.	.	.	.	.	.	.	.	.	.
**DE-19**	T	.	.	.	.	.	.	C	.	.	.	.	G	T	.	.	T	.	.	.	C	.	.	.	.	.	.	.	.	.	.	.	.	.	.	.	.	.	.
**DE-20**	T	.	.	.	.	T	.	C	.	A	.	.	G	.	.	.	T	.	.	T	.	.	.	.	.	.	.	.	C	.	.	.	.	.	.	.	.	.	.
**DE-21**	T	.	.	.	.	T	.	C	.	.	A	.	G	.	.	.	T	.	.	T	.	.	.	T	.	.	.	.	.	.	.	.	.	.	.	.	.	.	.
**DE-22**	T	.	.	.	.	T	.	C	.	.	.	.	.	.	.	.	T	.	.	T	.	.	.	.	.	.	.	.	.	.	.	.	.	.	.	.	.	.	.
**DE-23**	T	.	.	.	.	T	.	C	.	.	.	.	G	.	.	.	T	.	.	T	.	.	.	.	.	.	.	.	C	.	.	.	.	.	.	.	.	.	.
**DE-25**	T	.	.	.	.	T	.	C	.	.	.	.	G	.	.	.	T	.	.	T	.	.	.	.	.	.	.	.	.	.	.	.	.	.	.	.	.	G	.
**DE-24**	T	.	.	.	.	T	.	C	.	.	.	.	G	.	.	.	T	.	.	T	.	.	.	.	.	.	.	.	.	.	.	.	.	.	.	.	.	.	.
**DE-26**	T	.	.	.	.	T	.	C	.	.	.	.	G	.	G	.	T	.	.	T	.	.	.	.	.	.	.	.	.	.	.	.	.	.	.	.	.	.	.
**DE-27**	T	.	C	.	.	.	.	C	.	.	.	.	G	.	.	.	T	.	.	.	.	.	.	.	.	.	.	.	.	.	.	.	.	.	.	.	.	.	.
**DE-28**	T	.	C	.	.	.	.	C	.	.	.	.	G	.	.	.	T	.	.	.	.	.	.	T	.	.	.	A	.	.	G	T	.	C	.	.	.	.	.
**DE-29**	T	.	C	.	.	.	.	C	.	.	.	.	G	.	.	.	T	.	.	.	.	.	.	T	.	.	.	.	.	.	.	T	.	.	.	.	.	.	.
**DE-30**	T	.	C	.	.	T	A	C	.	.	.	C	G	.	.	.	T	.	.	T	.	.	.	T	.	.	.	.	.	.	.	T	.	.	.	.	.	.	.
**DE-31**	T	.	C	G	.	.	A	C	C	.	.	C	G	T	.	.	T	T	A	T	.	.	.	T	.	.	.	.	.	A	.	T	.	.	.	T	.	.	T
**DE-32**	T	.	T	G	.	.	A	C	C	.	.	C	G	T	.	.	T	.	.	T	.	.	.	T	.	.	.	.	.	A	.	T	.	.	.	.	.	.	.
